# Complete genome characterization of human noroviruses allows comparison of minor alleles during acute and chronic infections

**DOI:** 10.1099/acmi.0.000203

**Published:** 2021-02-17

**Authors:** Daniel Kelly, Khuzwayo C. Jere, Alistair C. Darby, David J. Allen, Miren Iturriza-Gómara

**Affiliations:** ^1^​ Institute of Infection, Veterinary and Ecological Sciences, University of Liverpool, Liverpool, UK; ^2^​ Malawi-Liverpool Wellcome Trust – Clinical Research Programme, College of Medicine, University of Malawi, Blantyre, Malawi; ^3^​ Centre of Genomic Research, Institute of Integrative Biology, University of Liverpool, Liverpool, UK; ^4^​ Department of Pathogen Infection Biology, Faculty of Infectious and Tropical Diseases, London School of Hygiene & Tropical Medicine, London, UK; ^5^​ Virus Reference Department, National Infections Service, Public Health England, Colindale, London, UK; ^6^​ NIHR Health Protection Research Unit Gastrointestinal Infections, Liverpool, UK; ^†^​ Present address: Department of Infection Biology, Faculty of Infectious and Tropical Diseases, London School of Hygiene & Tropical Medicine, London, UK

**Keywords:** norovirus, chronic infection, acute infection, enrichment, next generation sequencing, longitudinal sampling

## Abstract

Human noroviruses (HuNoVs) circulate globally, affect all age groups and place a substantial burden upon health services. High genetic diversity leading to antigenic variation plays a significant role in HuNoV epidemiology, driving periodic global emergence of epidemic variants. Studies have suggested that immunocompromised individuals may be a reservoir for such epidemic variants, but studies investigating the diversity and emergence of HuNoV variants in immunocompetent individuals are underrepresented. To address this, we sequenced the genomes of HuNoVs present in samples collected longitudinally from one immunocompetent (acute infection) and one immunocompromised (chronic infection) patient. A broadly reactive HuNoV capture-based method was used to concentrate the virus present in these specimens prior to massively parallel sequencing to recover near complete viral genomes. Using a novel bioinformatics pipeline, we demonstrated that persistent minor alleles were present in both acute and chronic infections, and that minor allele frequencies represented a larger proportion of the population during chronic infection. In acute infection, minor alleles were more evenly spread across the genome, although present at much lower frequencies, and therefore difficult to discern from error. By contrast, in the chronic infection, more minor alleles were present in the minor structural protein. No non-synonymous minor alleles were detected in the major structural protein over the short sampling period of the HuNoV chronic infection, suggesting where immune pressure is variable or non-existent, epidemic variants could emerge over longer periods of infection by random chance.

## Introduction

Human noroviruses (HuNoVs) are the main causative agents of acute gastroenteritis in the UK [1, 2], and are a leading cause of non-bacterial gastroenteritis globally [3, 4], responsible for 18 % of all cases of gastroenteritis [5]. The genus *Norovirus* of the family *Caliciviridae* comprises a group of viruses with a single-stranded positive sense RNA genome of approximately 7500 nt in length. The HuNoV genome is organized as three ORFs; ORF1 encodes the non-structural proteins (NS1–7); ORF2 encodes the major capsid protein, VP1 – itself arranged as conserved S, P1 and the hypervariable P2 domains; and ORF3 encodes the minor capsid protein (VP2) [6]. The genus *Norovirus* is further organized as genogroups (G) and HuNoVs are classified into GI, GII and GIV, within which there are nine, 27 and two VP1 types or 14, 37 and one polymerase types, respectively [7]. The GI and GII HuNoVs are responsible for most of the human infections observed, but GII, and in particular GII genotype 4 (GII.4), HuNoVs are the most commonly detected by VP1 typing worldwide [8].

The GII.4 HuNoVs are further subdivided into strains based on ORF2/VP1 sequence diversity, which have emerged as successive antigenically distinct viruses with 2- to 3-year periodicity [9–11] from the 2000s to 2012. Through the 2010s, different genetic strains of GII.4 viruses have been found co-circulating globally, all with Sydney/2012 ORF2 sequences but different RNA-dependent RNA polymerase (RdRp/NS7) types [12, 13].

This diversity, which is observed across the genus *Norovirus*, is driven by two mechanisms: first, the error-prone viral RdRp, which during genome replication induces genetic changes that can in turn create antigenic drift; and secondly, further diversity is generated by recombination, particularly at the ORF1/2 junction [9–11, 14, 15].

Surveillance systems have typically used Sanger sequencing to determine partial consensus sequence of the RdRp or VP1 for molecular epidemiology studies, but these approaches are susceptible to false-negative results where primer mismatches occur, do not resolve the complete genome and are limited in their resolution of minor variants. Massively parallel sequencing (MPS), or *de novo* unbiased sequencing technologies, have superseded Sanger sequencing as these offer higher sensitivity, whole genome reconstruction and an ability to survey virus population diversity within the host [16].

Pathogen nucleic acids, particularly viral RNA, represent a very small proportion of the total nucleic acids present in a clinical sample [17]. Therefore, it is necessary to enrich the target nucleic acid against host, bacterial and other viral nucleic acids prior to library preparation and MPS. Methods using virus filtration [18], RNA poly-A tail selection [19–21], RNA bait selection [22], rRNA depletion [23, 24], virus ultracentrifugation [25] or porcine gastric mucin (PGM) magnetic bead (MB) capture [26] have been applied to clinical (faecal) samples to enrich norovirus RNA for subsequent MPS.

Studies of HuNoV have investigated chronic and, to a lesser extent, acute infection using MPS [24, 25, 27, 28]. Bull *et al*. demonstrated the presence of a highly heterogenous population of HuNoV minority variants over the course of a chronic infection [27], and in a separate study, using similar MPS technology, Kundu *et al*. identified an excess of fixed synonymous mutations occurring during chronic HuNoV infection [28].

In contrast, observation of virus intra-host variation during infection in an immunocompetent host showed HuNoV minority variants are more homogenous over the course of infection, with a single minority variant detected in the ORF2/3 region [27].

In this study, PGM-MBs were used as an enrichment methodology to recover HuNoV from clinical specimens prior to MPS. This method was applied to clinical samples collected longitudinally from two patients: one acute HuNoV infection, collected through to convalescence; and one chronic HuNoV infection. HuNoV sequences from these patients were analysed using a novel bioinformatics pipeline, to determine the presence of minority variants within he host, and to monitor which sites in the virus genome could be under selective pressure.

## Results

### Validation of PGM-MB capture as a tool for HuNoV recovery prior to MPS

The effect on enrichment from use of either PGM-MB capture or including additional PCR cycling upon the number of HuNoV reads recovered for downstream analysis was investigated by applying these treatments to clinical samples with a high (Sample A) and a low (Sample B) viral genome copy number.

**Fig. 1.**
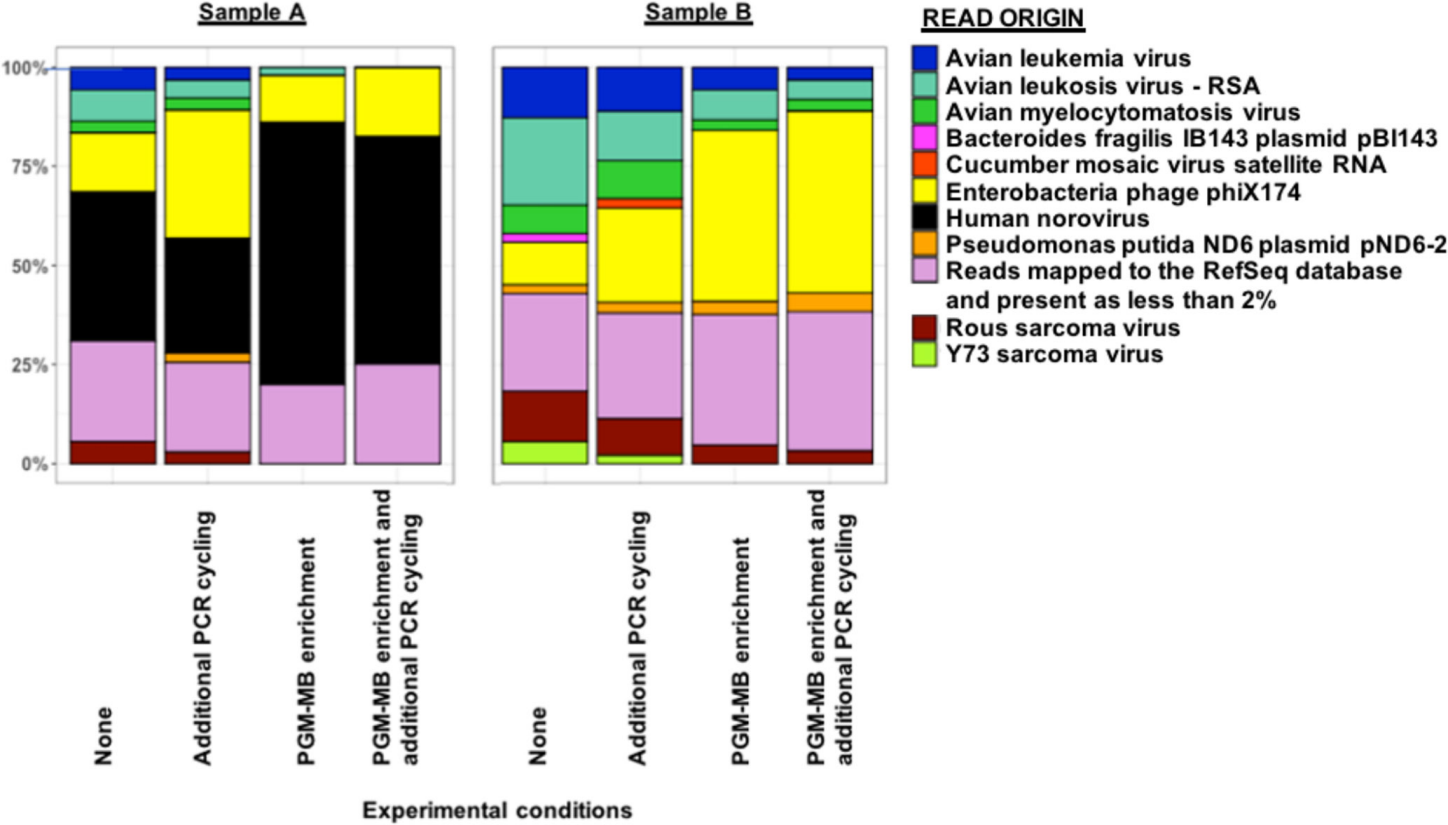
Relative abundance of MPS reads (Illumina) mapped from two GII HuNoV clinical samples. Sample A (high viral genome copy number/g) and sample B (low viral genome copy number/g), with or without a combination of PGM capture and additional PCR cycling.

At high starting viral genome copy number (Sample A), the greatest enrichment effect was observed after PGM-MB capture, whilst a combination of additional PCR cycling and PGM-MB capture led to a lower GII HuNoV read recovery (Fig. 1). A decrease in the proportion of HuNoV reads was detected if additional PCR cycling was performed in comparison to no treatment (Fig. 1). The increases in OTRs without normalization by genome size for sample A upon PGM-MB capture, PCR cycling or both treatments were 0.89%, −0.06 % or 0.65%, respectively.

At low starting viral genome copy number (Sample B), HuNoV reads ranged from 0.002 to 0.01% and therefore PGM-MB capture, additional PCR cycling or both treatments were unable to enrich the proportion of HuNoV reads above 2 % (Fig. 1).

### Characterization of virus diversity during acute infection

HiSeq (Illiumina) reads from patient P1 were *de novo* assembled into contiguous sequences, and a GII.P4 (New Orleans 2009)/GII.4 (Sydney 2012) HuNoV (Table 1) was identified. The total number of reads from samples collected on days 3, 5, 6, 10 and 11 ranged from 6.2 to 14×10^6^, 9.0 to 20×10^6^, 2.9 to 61×10^6^, 8.8 to 15×10^6^ and 6.8 to 19×10^6^, respectively, and the percentage of OTRs against the consensus sequence ranged from 0.01 to 0.25 %, 0.03 to 0.43 %, 0.03 to 0.52 %, 0.01 to 0.23 % and 0.00 to 0.03%, respectively.

**Table 1.** Quantification of GII HuNoV in archived and collected clinical samples by qPCR (one case was severely immunocompromised, P2, whilst no known immunodeficiencies or immunosuppressive therapy were present for P1)

Patient ID	Immunosuppresive comorbidity	Genotype	Year of detection	Day collected post-recruitment	GII HuNoV genome copies/qRT-PCR
P1	None	GII.4	2012	3	3.23×10^7^
				5	2.94×10^7^
				6	1.22×10^7^
				12	5.91×10^5^
				15	1.25×10^4^
P2	Haemopoetic stem cell transplantation	GII.7	2012	3	1.03×10^9^
				9	1.29×10^9^
				12	1.54×10^8^
				15	1.29×10^9^

The aligned sequence data were too fragmented for inferences of consensus change if the percentage of the genome recovered was below 40% and of insufficient coverage to call minority variants if the median of a replicate was <100. Therefore, three replicates were excluded from inferences of consensus change (one each from day 3, 10 and 11) and minority variants were not called from eight replicates (one each from day 3, 5 and 6 and all replicates from days 10 and 11).

Complete or near complete genomes were recovered from all samples except for the latest time point (day 11) of patient P1 (Table S1, available in the online version of this article). At the latter time points (days 10 and 11), HuNoV genomes were recovered at low median coverage, which coincided with a decrease in virus genome copies/qRT-PCR (Tables 1 and S1).

Two synonymous consensus mutations were inferred at positions 5323 and 5431 of ORF2 (S domain of VP1) in the virus sequence during infection (Table 2). On days 3, 5 and 6, the consensus allele was thymine and guanine at position 5323 and 5431, respectively (Table 2). Although minority alleles were detected on day 6, the allele switching point for position 5323 (T>C) and 5341 (G>A) appeared to occur on day 10, which subsequently became fixed on day 11 (Table 2).

**Table 2.** Consensus genome changes observed in longitudinal samples from patients P1 and P2

		Patient P1								Patient P2			
Day of sample collection post-recruitment	Replicate	Nucleotide frequency at position (%)											
		5323				5431				725			
		A	C	G	T	A	C	G	T	A	C	G	T
3	1				100			100			25		75
	2				100			100			45		55
	3										43.1		56.9
5	1				100			100					
	2	0.2	0.2		99.6			99.8					
	3				100			100					
6	1				100			100					
	2		0.2		99.8	0.1	0.1	99.8					
	3		9.8		90.2	3.6		96.4					
9	1										60.6		39.4
	2										43.5		56.5
	3										61.3		38.7
10	1		70		30	21.1		78.9					
	2		0		100	0		100					
11	1		100			100							
	2		100			100							
12	1										64.1		35.9
	2										33.3		66.7
15	1										66.7		33.3
	2										55.8		44.2
	3										72.7		27.3

### Characterization of virus diversity during chronic infection

HiSeq (Illumina) reads from patient P2 were assembled in the same manner to identify a GII.P7/GII.7 HuNoV (Table 1). The total number of reads from samples collected on days 3, 9, 12 and 15 ranged from 0.55 to 23×10^6^, 2.0 to 17×10^6^, 0.0023 to 22×10^6^ and 0.47 to 13×10^6^, respectively, and the percentage of OTRs against the consensus sequence ranged from 0.13 to 0.23 %, 0.14 to 0.21 %, 0.09 to 0.12 % and 0.16 to 0.85 %, respectively.

To infer consensus changes, replicates were filtered in the same manner as described above. However, replicates were filtered for minority variant calling if the median was >10, which was less stringent due to a smaller range of medians and a higher minimum median. Therefore, one replicate (day 12) was excluded from inferences of consensus change and minority variant calling due to a failed library preparation (Table S1, Fig. S2).

Complete or near complete genomes were recovered at all time points, which coincided with shedding of consistently high virus titres (Tables 1 and S1, Fig. S2). Despite variability in median coverage between replicates originating from patient P2, three-quarters had a median which was at least 40-fold or above (Table S1).

A synonymous mutation was inferred at position 725 (p48 of the non-structural proteins) in the GII.7 HuNoV sequence during infection (Table 2). On day 3 the dominant allele at position 725 was thymine, and then on days 9 and 12 a mixture of cytosine and thymine existed, but by day 15 the dominant allele in each replicate at position 725 was cytosine (Table 2).

### Comparison of minority variants detected during an acute versus a chronic infection

**Fig. 2.**
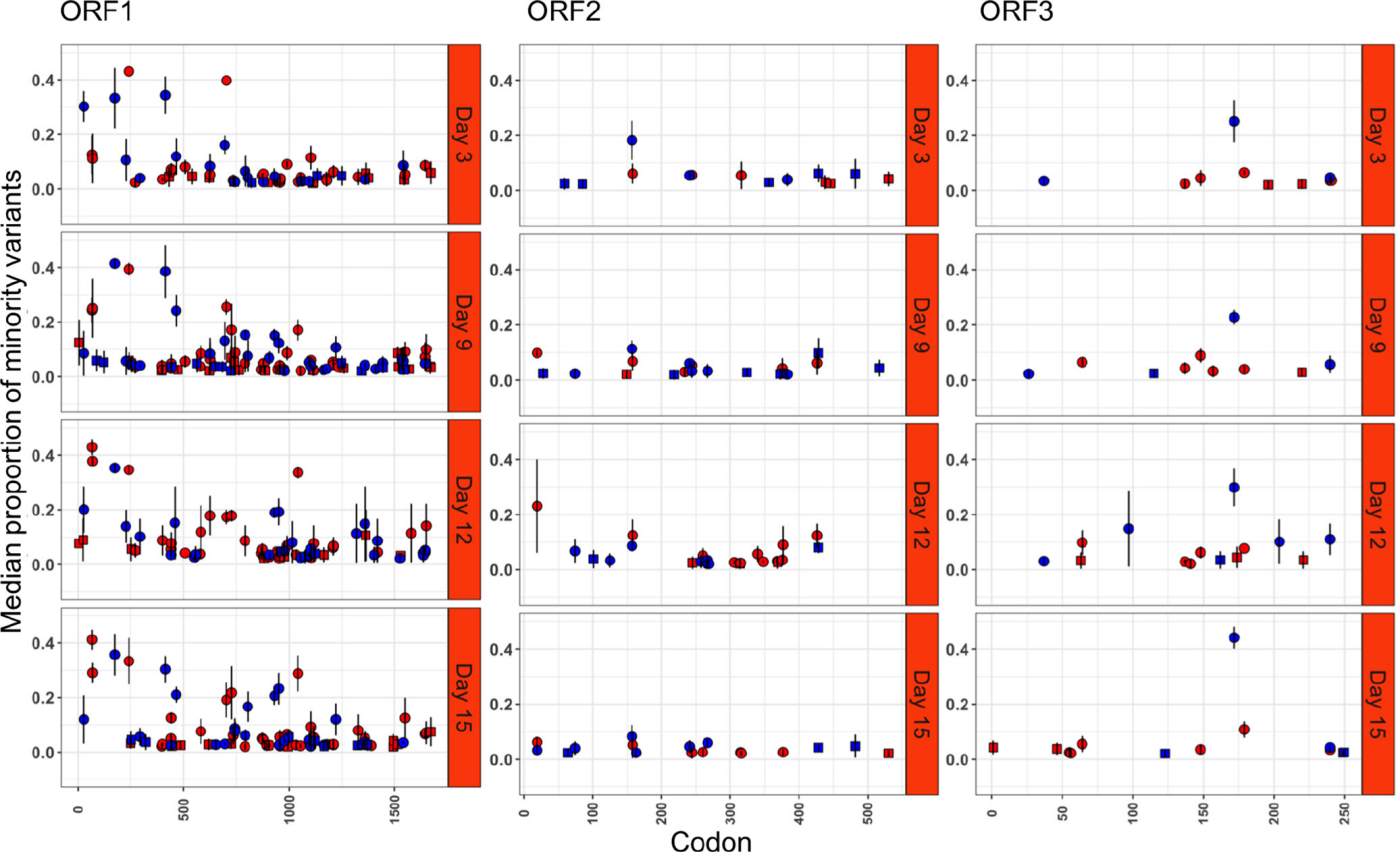
Proportions of minority variants identified in samples collected from patient P2 (red=non-synonymous, blue=synonymous, square=non-persistent and circle=persistent).

**Fig. 3.**
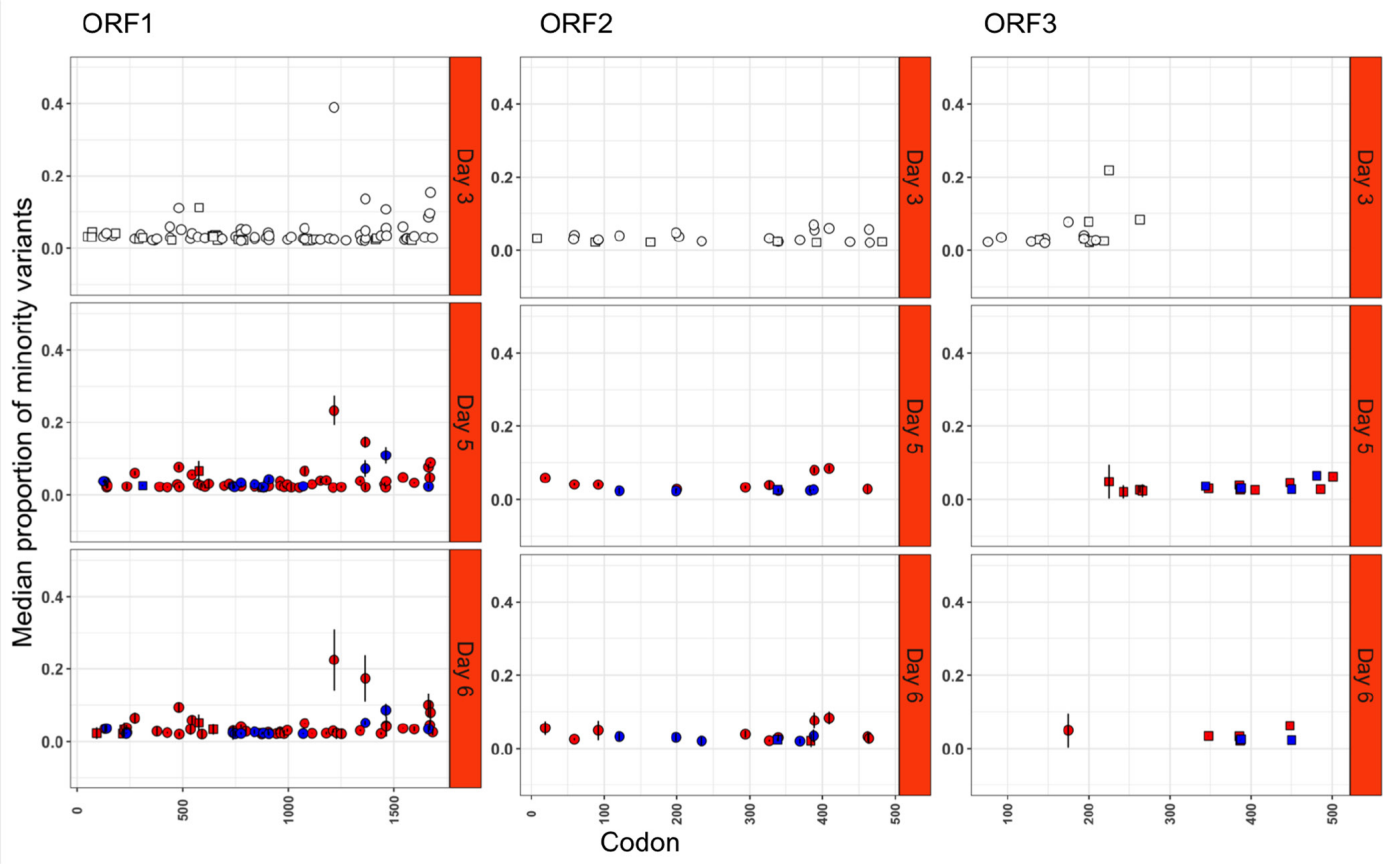
Proportions of minority variants identified in samples collected from patient P1 (white=single observation, red=non-synonymous, blue=synonymous, square=non-persistent and circle=persistent).

Minority variants were observed in more genes across the complete HuNoV genome, after normalization by ORF size, during chronic infection, compared to acute infection (Figs 2 and 3). More persistent minority variants existed during the chronic infection than the acute infection (306 versus 243), and these occupied a significantly greater fraction of the virus population than non-persistent minority variants (*P*<0.05). During chronic infection, it was noted that synonymous mutations occupied a significantly greater fraction of the population in ORF1 (Fig. 2) when compared to ORF2 (*P*<0.05), but at later time points from days 12 to 15 the non-synonymous minority variant fraction decreased significantly across the genome (*P*<0.05).

**Fig. 4.**
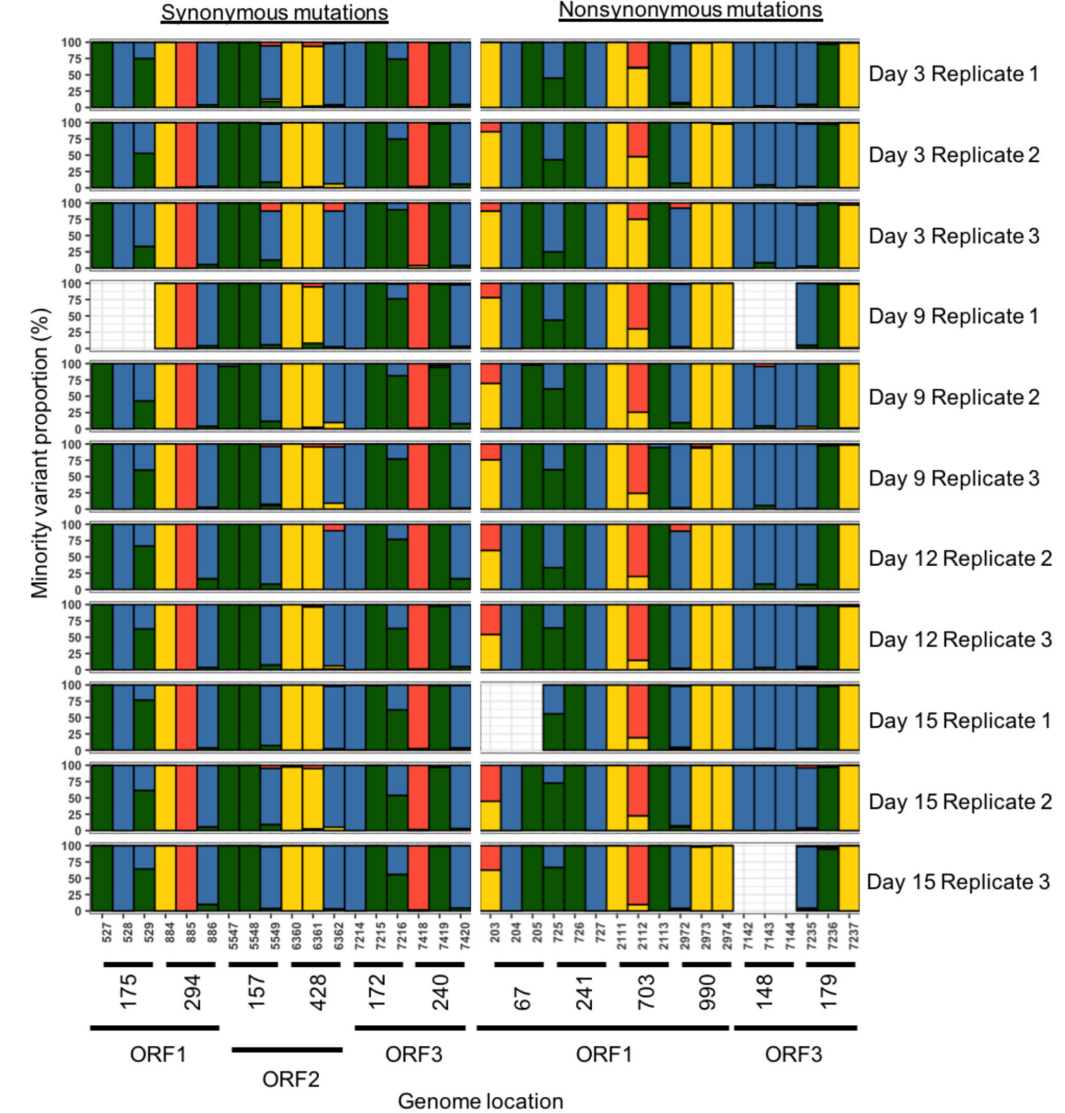
Variant content of conservatively identified codon positions in samples collected from patient P2 (adenine=red, cytosine=green, guanine=yellow and thymine=green).

If a minority variant was observed in all replicates analysed from the chronic infection at a single time point, there was a tendency for it to be persistent (Fig. 2). In total, 12 persistent minority variants were detected of which half were non-synonymous (Fig. 4). The non-synonymous mutations were present in ORF1 (4/6), and the remainder present in ORF3 (2/6). Overall, non-synonymous mutations were transitions relative to the consensus sequence. For codon positions 67 and 990 of ORF1 and 179 of ORF3, the presence of a persistent minority variant was not in complete agreement amongst all replicates and time points, being observed in 10/11, 9/11 and 8/11 sequences, respectively.

In contrast, during chronic infection, synonymous persistent minority variants were distributed evenly across the three ORFs (Fig. 4). Overall, synonymous mutations were transitions relative to the consensus sequence with the exception of position 428 (ORF2). The persistent minority variant at position 428 was in agreement 6/11 times across replicates and time points and was the only synonymous mutation without complete agreement.

**Fig. 5.**
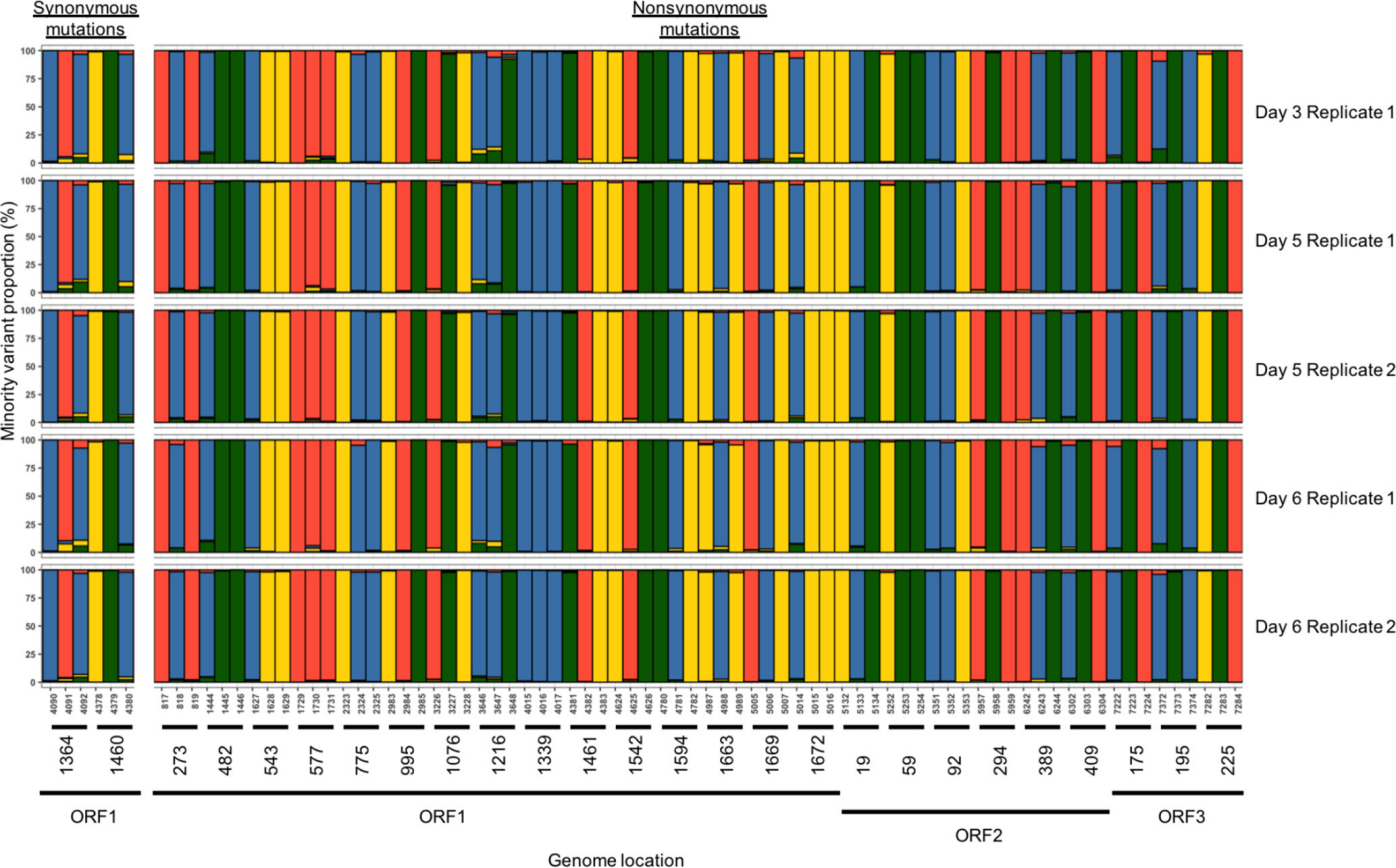
Variant content of conservatively identified codon positions in samples collected from patient P1 (adenine=red, cytosine=green, guanine=yellow and thymine=green).

During acute infection, 26 persistent minority variants were detected in acute infection of which 24 were non-synonymous (Fig. 5). The highest number of non-synonymous minority variants was detected in ORF1 (15/24), followed by ORF2 (6/24) and ORF3 (3/24) (Fig. 5). During acute infection, 2/26 codon positions had a homogeneous minority variant population, and minority variants were present at a significantly lower proportion overall in comparison to chronic infection (*P*<0.05).

### Cross-validation of a novel bioinformatics pipeline

**Fig. 6.**
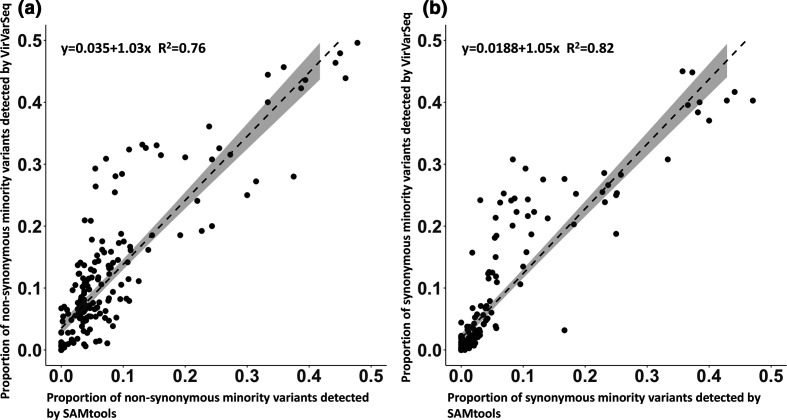
A linear regression model comparing the synonymous (**a**) and non-synonymous (**b**) persistent minority variants detected by VirVarSeq versus SAMtools in patient P1 and patient P2 (shaded region=0.95 confidence interval).

To validate the novel bioinformatics pipeline and the minor variants detected, we also conducted a comparison against an established minor variant calling tool (VirVarSeq). The output of VirVarSeq was filtered for infection-specific codon positions (Figs 4 and 5) and a pairwise analysis demonstrated a positive correlation between the proportions of synonymous (*R* ^2^=0.82) and non-synonymous (*R* ^2^=0.76) minority variants detected (Fig. 6).

## Discussion

Here we demonstrate that using PGM-MBs as an enrichment tool for HuNoV from faecal specimens prior to MPS enhances recovery of near-complete viral genomes. A novel bioinformatics pipeline determined intra-specimen changing or static codon positions during acute and chronic infection.

Even during acute symptomatic viral infection, virus nucleic acids can represent a very small proportion of the total genetic material in a clinical specimen [17], and overcoming this is essential in obtaining high-quality sequence data. A number of approaches have been applied to address this problem, although each also presents challenges: for example targeting the poly-A tail of genomic RNA [19–21] can enrich both viral and non-viral nucleic acids; alternatively, targeting multiple regions of a virus genome with a family of short oligonucleotides [22] can be affected by primer biases. Furthermore, PGM-MBs can concentrate enteric viruses, including norovirus, from environmental samples prior to MPS [26]. Here, we used a similar PGM-MB approach, with MPS, to analyse longitudinally collected clinical specimens, which allowed differences in the virus genome to be accurately identified in specimens where a low concentration of the target of interest was present relative to the total nucleic acid. Furthermore, a novel bioinformatics pipeline was developed and validated against existing tools, and this enabled consensus genome differences and sets of persistent minority variants to be identified between replicates and time points.

This methodology has some limitations: PGM-MB is not HuNoV-specific, so other pathogens or microorganisms in the specimen may be captured, creating competition for binding sites on the beads. Also, robust analysis requires sampling across different time points during infection, and this can be difficult to achieve in clinical and public health settings, in particular for acute infections which quickly proceed to the convalescent phase and virus shedding begins to decline. This could explain why persistent minority variants were present at a much lower frequency in acute infection, and therefore difficult to discern from background signal. The persistent minority variants were present at more positions than chronic infection and could be due to the analysis of more replicates and time points in chronic infection, which meant more stringent criteria during filtering.

During acute infection, 26 minority variants were detected (days 3–6) in all regions of the virus genome except the P1 subdomain of ORF2 and were present at an average frequency of 4.2 %. We also observed two synonymous mutations of the consensus sequence that occurred at ORF2 over 11 days. In agreement with Bull *et al*. [27], we found allele frequencies to be more stable in comparison to chronic infection.

In comparison, a single synonymous mutation of the consensus sequence occurred at ORF1 over 15 days of a chronic infection, which is within the range described by van Beek *et al*. (0.03–0.37/day) [29]. In total, 12 minority variants were detected during chronic infection (days 3–15), which were present at an average frequency of 16.6 % (range 1.4–42.9 %), again similar to that described by Bull *et al*. (2.1–59.5 %), although described in a chronic infection over a longer period of 288 days [27].

The proportions of synonymous persistent minority variants were significantly greater in ORF1 than in ORF2 across the whole time course of the chronic infection, and all ORF1 synonymous variants were located in the non-structural protein p48. This could mean codon positions 175 and 294 are restricted to synonymous changes only, as any other change would have an inhibitory effect upon the function of p48, whether that be disrupting intracellular protein trafficking or another role [30].

Non-synonymous minority mutations were detected in the ORF1 non-structural proteins p48, p22 and VPg, and in VP2-encoding ORF3. In p48, p22 and VP2, the presence of positive selective pressure (fixation of non-synonymous mutations) in acute and chronic infection has been described previously [24, 25, 31, 32]. The non-structural protein, p22, and structural protein VP2 are likely to influence the replication process with roles in antagonism of protein secretion [33] and host cell entry [34], respectively. It is notable that none of the non-synonymous minor variants observed during chronic infection were found in ORF2, given the breadth of literature reporting minor variants or amino acid fixations in epitopes or putative epitope sites in VP1 during chronic infection [24, 29, 35, 36]; however, these studies sampled over a much longer period (76–1811 days). Distinct antigenic types of HuNoV have been reported to emerge over the course of chronic infection, leading to predictions that changes occurring in immunodominant epitopes could lead to evasion of population humoral immunity, and subsequent strain emergence [37, 38]. However, non-static codon sites of the non-structural proteins which are not exposed to antibody recognition could have an important role in innate immunity as T cell epitopes. Peptide libraries designed from the proteins of a GII.P31/GII0.4 (Sydney) genome were demonstrated to stimulate and subsequently induce IFN-γ activity in donor T cells; however, peptides from p48 were least effective and those from VP2 and p22 ranked second and fourth after VP1, respectively [39].

Comparisons made between the acute and chronic infection were limited by the presence of two separate genotypes under different levels of immune pressure, and therefore it was difficult to ascertain whether observations are related to the environment or the virus itself.

Minority variant detection in repeated or longitudinal sampling will be a useful tool to examine the effects of selective pressure between seeding events and outbreaks (transmission bottlenecks), within *in vitro* models or *in vivo* models (e.g. as part of vaccine or antiviral development) [40–42]. This tool will detect minority variants which are conserved between replicates, or more widely between time points, and therefore the conservation of minor alleles can be used to link seeding events and outbreaks. The accurate identification of minor alleles within replicates before and after the induction of selective pressure (antiviral or antibody) can provide insights into novel mechanisms of antiviral resistance or how HuNoV fitness may change within and between hosts, over the duration of infection.

## Methods

### Preparation of PGM-MBs

MagnaBind carboxyl derivatized beads (Fisher Scientific) were prepared as described in the manufacturer’s instructions, pooled and stored at 4 °C. PGM III (Sigma) was dissolved in conjugation buffer to a concentration of 7.5 mg ml^−1^ for the coupling reaction.

### Clinical sample collection, preparation and characterization

Norovirus-positive inpatients with symptoms of acute gastroenteritis were approached for consent to participate in the study and provide longitudinal faecal samples (Table 1).

All stool samples were prepared as 10 % (v/v) suspensions in sterile PBS (Sigma). Stool suspensions were centrifuged at 12 470 ***g*** for 10 min to separate particulate matter from supernatant.

For initial optimization, two anonymized stool specimens, known to be HuNoV-positive, were used: Sample A (2.7×10^9^ viral genome copy number/g) and Sample B (1.2×10^4^ viral genome copy number/g) were prepared for experiments to optimize PGM-MB enrichment.

For the main study, longitudinal specimens were collected from two patients. Patient 1 (P1) had an acute HuNoV infection, from whom five stool specimens (1.25×10^4^–2.94×10^7^ viral genome copies/qRT-PCR) were collected across 15 days from enrolment. Patient 2 (P2) had a chronic HuNoV infection, from whom four stool specimens (1.54×10^8^–1.29×10^9^ viral genome copies/qRT-PCR) were collected across 11 days from enrolment (Table 1).

### Optimization of HuNoV capture from clinical stool samples

For each optimization experiment, 100 µl of homogenous PGM-MBs were added to 1 ml of capture mixture containing stock stool suspension supernatant and sterile 0.1 M citric acid-sodium citrate buffer (Sigma) in a ratio of 1 : 3. The mixture was kept under constant mixing on a rotator shaker for 15 min. After mixing the PGM-MBs were recovered using a DynaL magnetic separation rack (Fisher Scientific) and washed with 1 ml 0.1 M citric acid-sodium citrate buffer three times. The PGM-MBs were then eluted into 60 µl sterile PBS (Sigma) and transferred to a sterile microcentrifuge tube.

### Nucleic acid recovery and DNase treatment

Nucleic acids were extracted from the PGM-MB eluate by a guanidium isothiocyanate silica method [43]. The extracted RNA was treated with DNase I (Sigma) and purified from solution using the QIAquick Gel Extraction kit (Qiagen), following the manufacturer’s instructions.

### One-step quantitative PCR of GII HuNoV RNA

Quantitative (Taqman) real-time RT-PCR (qRT-PCR) was performed on a Rotor Gene 6000 (Qiagen) using the Rotor-Gene Multiplex RT-PCR Kit (Qiagen). The qRT-PCR targeted the ORF1/2 junction of the GII HuNoV genome, based on the assay described by Kageyama *et al*. [44], and was used to monitor enrichment and library preparation steps. Briefly, each final reaction contained 1× Rotor-Gene Multiplex RT-PCR Master Mix (Qiagen), 0.2 µl Rotor-Gene RT Mix (Qiagen), 1 µM each COG 2F/COG 2R primers (Sigma), 0.5 µM Ring 2 probe (5′ FAM/TAMRA 3′; Sigma), 5 µl of RNA sample and DEPC-treated water (Fisher Scientific) to a total volume of 20 µl. Quantitative real-time RT-PCR cycling conditions were as follows: 50 °C for 15 min, 95 °C for 2 min, and then 45 cycles of 95 °C for 15 s and 56 °C for 60 s. Fluorescence was detected at the 56 °C extension step. Absolute genome copy numbers in a qRT-PCR were extrapolated from an RNA standard. The RNA standard was produced by T7 *in vitro* transcription of the GII HuNoV ORF1/2 junction present in pCRGII3-3.

### MPS library preparation

HuNoV RNA was denatured at 95 °C before library preparation. Libraries were prepared using the ScriptSeq v2 RNA-Seq Library Preparation Kit (Illumina) according to the manufacturer’s instructions. At the amplification stage, Illumina-compatible barcodes replaced the reverse primer, to allow for sample multiplexing. Prior to sequencing on the HiSeq2500 Illumina platform at the Centre for Genomic Research, University of Liverpool, library size and concentration were analysed with the 2100 Bioanalyzer (Agilent Technologies) and Qubit dsDNA High Sensitivity assay (Fisher Scientific) respectively. A sample with a flat 2100 Bioanalyzer trace (non-detectable amount of library) after 12 cycles of non-specific PCR amplification was amplified for a further 10 cycles. If additional amplification was performed, amplicons were purified with AMPure beads (Beckman Coulter) at 0.7× the volume of the total PCR, to minimize the presence of primer dimer.

In total, two sets of library preparations were prepared for each sample. The first set of library preparations were sequenced on flow cell 1. The sequencing process was then repeated for set 1 in combination with set 2 on flow cell 2.

### Data analysis

HiSeq (Illumina) data were processed using the software (Table S2) and pipeline described (Fig. S1). HiSeq (Illumina) data were *de novo* assembled into contigs which became input for a basic local alignment. Alignments identified a suitable reference sequence for each patient, which was then used to derive a consensus sequence for each sample replicate. Consensus sequences for each patient and time point were deposited in GenBank (accession numbers: MW284776–MW284784) and became input for the Norovirus Automated Genotyping Tool to identify polymerase and capsid types [45]. For each patient, the replicate with the highest percentage of the consensus genome recovered was used for further read alignment (Table S1). The percentage of on target reads (OTRs) from alignment was calculated by Qualimap software version 2.2.1, available online at: qualimap.bioinfo.cipf.es [46].

To identify a consensus sequence change or minority variant at one time point, in patient P1 or P2, complete agreement between all library preparation replicates was necessary.

A series of novel R scripts were developed as a pipeline to perform the following analyses: (i) to generate frequencies of synonymous and non-synonymous mutations from the SAMtools output, which had been converted to a tabular format in Perl; (ii) to assess the correlation in minority variant calling between the pipeline described in this paper and VirVarSeq [47] under default settings; and (iii) to detect minority variants, in a conservative manner, across different replicates of a longitudinal data set.

Analyses were performed to identify the specific locations of mutations, and whether minority variants persisted in chronic GII.7 and acute GII.4 infection. A minority variant was defined as a consensus mismatch present in all replicates at one time point, and if this was present at multiple time points it was defined as a persistent minority variant. Persistent minority variants were filtered for further analysis if their interquartile range (IQR) across replicates overlapped between sequential time points and their lower quartile exceeded the time point population median multiplied by 10 (Figs S3–S6). Statistical analyses were performed with the Wilcoxon Rank Sum Test in R under default settings. To remove background noise from the analysis, the data sets from patients P1 and P2 were filtered to remove consensus differences which existed as 2 % of the minority variant population or below.

### Metagenomic analysis

To compare the effectiveness of the PGM treatment for HuNoV enrichment metagenome analysis approaches were used. The Genome Relative Abundance and Average Size (GAAS) software version 0.17, available online at: https://sourceforge.net/projects/gaas/files/gaas/GAAS-0.17/ [48], was used for this purpose. First, fastq files were subsampled to a range of 2–4×10^6^ reads per sample with the reformat shell script available in the BBMap tool suite software version 37.53, available online at: http://sourceforge.net/projects/bbmap/, and subsequently converted to fasta format with Seqtk software version 1.2, available online at: https://github.com/lh3/seqtk/. Similarities in each fasta file were examined against a RefSeq database (2012) for protozoa, microbes and viruses under the nucleic similarity filtering option. The database was manipulated to add HuNoV consensus sequences from each positive stool sample. The GAAS software returned OTRs as a percentage of the total number of reads with or without normalization by reference genome size.

## Supplementary Data

Supplementary material 1
