# Supplementary material for: Complete genome characterization of human noroviruses allows comparison of minor alleles during acute and chronic infections
Source: Access Microbiol. 2021 Feb 17;3(3):000203. doi: 10.1099/acmi.0.000203 (PMC8209700; doi:10.1099/acmi.0.000203)

Supplementary Table 1

Patient	Day	Replicate	Coverage				Percentage of genome recovered (%)
			Median	Lower quartile	Upper quartile	IQR	
P1	3	1	136	95	183	88	99.8
		2	5	3	7	4	80.8
	5	1	323	234	435	201	100.0
		2	513	393	687	294	99.9
		3	8	4	13	9	94.7
	6	1	139	97	188	91	99.9
		2	28	19	36	17	99.2
		3	892	654	1205	551	99.9
	10	1	5	2	9	7	77.8
		2	10	6	17	11	96.8
	11	1	1	1	2	1	13.0
		2	3	2	6	4	73.8
		3	2	2	3	1	49.1
P2	3	1	14	9	22	13	97.7
		2	54	37	80	43	99.8
		3	86	62	148	86	99.8
	9	1	45	33	68	35	99.9
		2	56	39	86	47	99.9
		3	53	32	81	49	100.0
	12	1	189	137	273	136	99.8
		2*					0.3
		3	13	8	20	12	98.9
	15	1	40	28	56	28	99.8
		2	161	114	245	131	99.8
		3	41	28	66	38	99.8

Supplementary Table 2

Software name	Version	Function	Reference
SPAdes	v.3.5.0	De novo assembly	(Bankevich *et al.*, 2012)
Blastn	2.2.27+	Reference search	(Zhang *et al.*, 2000)
Trimmomatic	0.36	Quality control	(Bolger, Lohse and Usadel, 2014)
FastQC	V0.10.1	Diagnostics	(Andrews, 2010)
Burrows-Wheeler Aligner	0.5.9-r16	Aligner	(Li and Durbin, 2009)
SAMtools	0.1.18-r580	Sequence alignment map processing and variant calling	(Li *et al.*, 2009)
Picard tools	2.1.1	Remove duplicate reads	Available online at: http://broadinstitute.github.io/picard/
BBMap	37.53	Subsample reads	Bushnell B. BBMap short-read aligner, and other bioinformatics tools. 2016. http://sourceforge.net/ projects/bbmap/.

Supplementary Figure 1

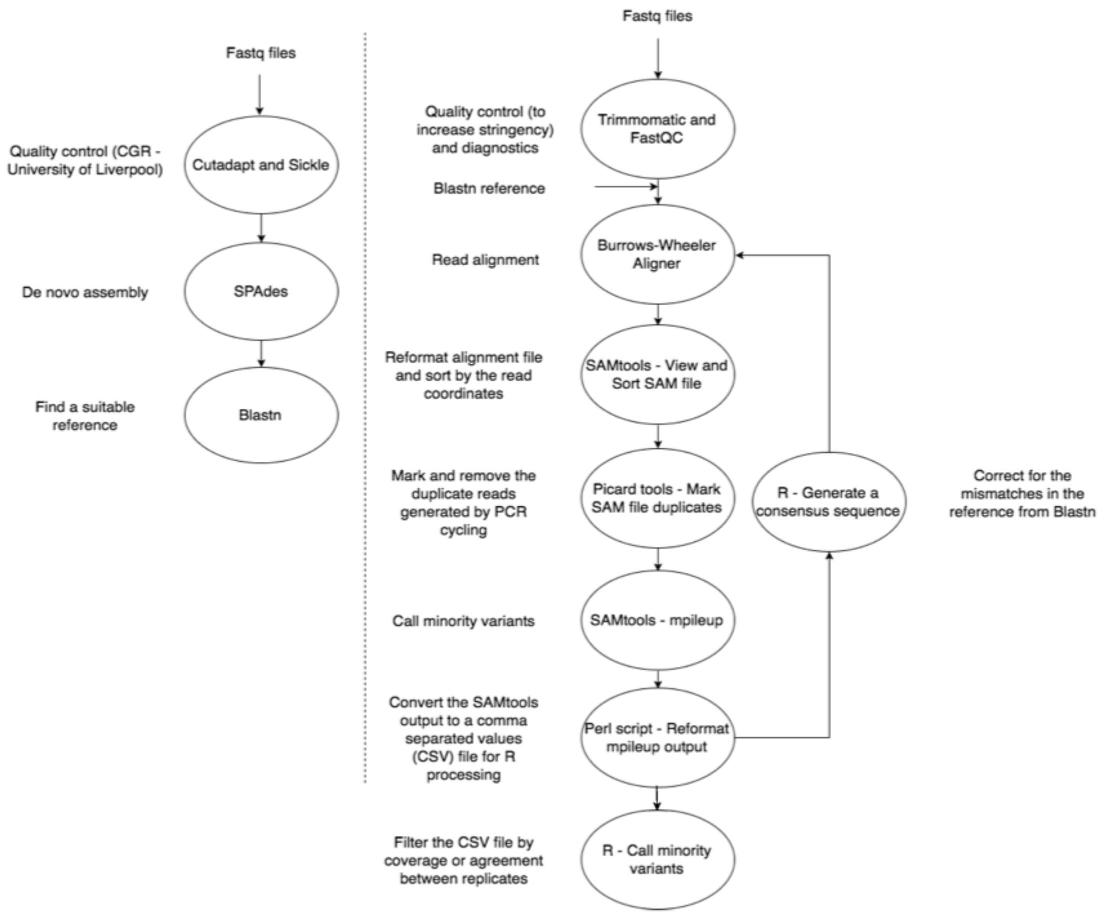

Supplementary Figure 2

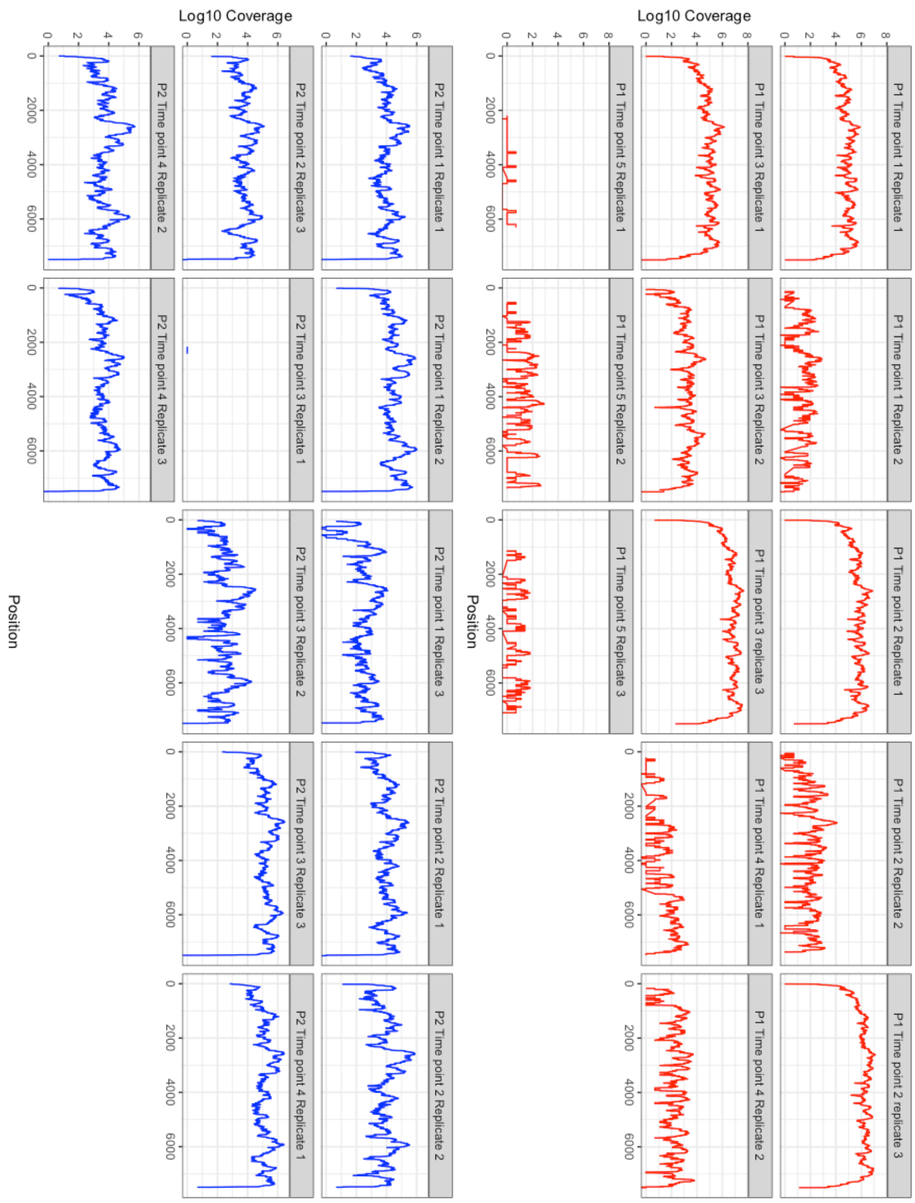

Supplementary figure 3

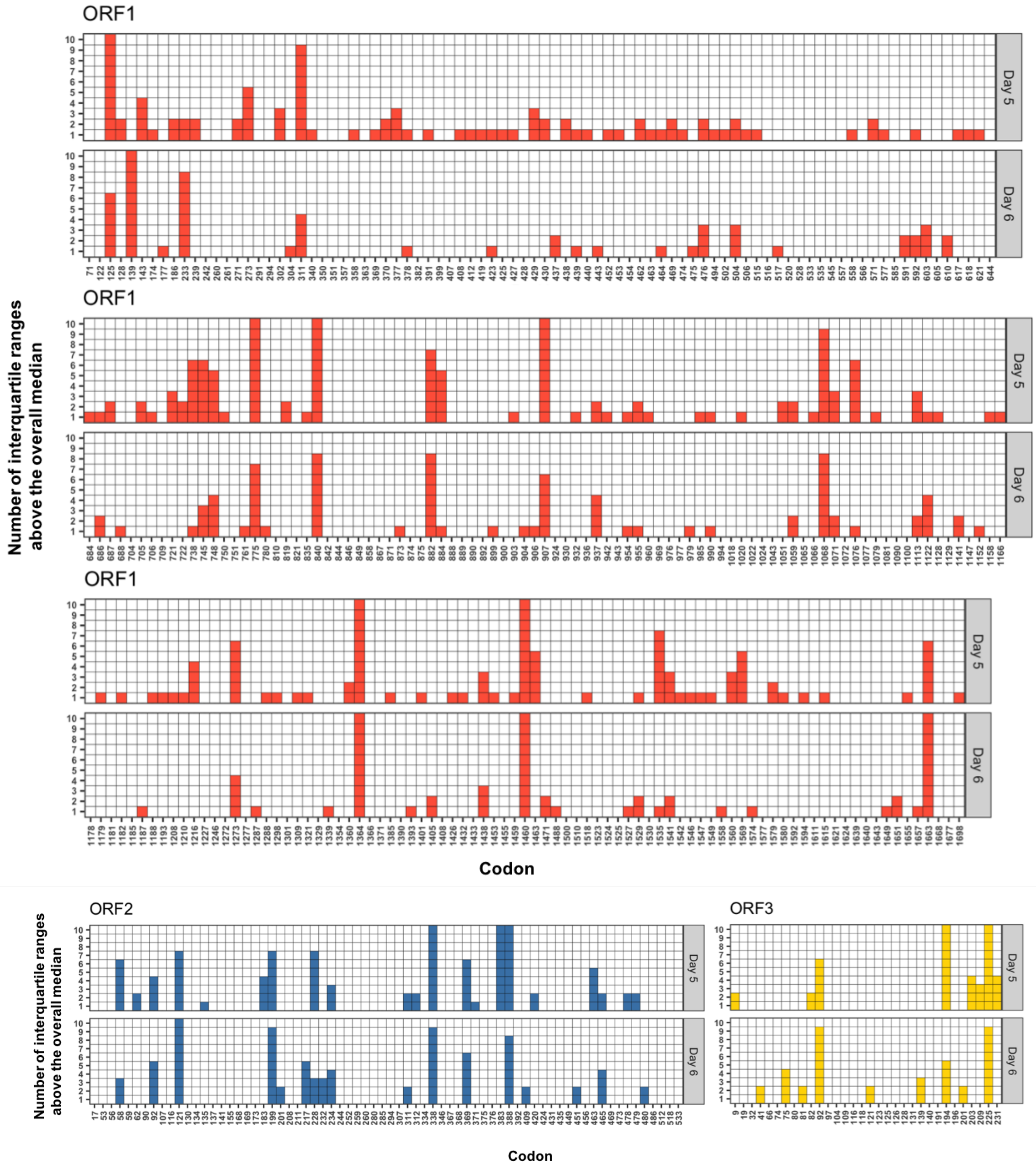

Supplementary Figure 4

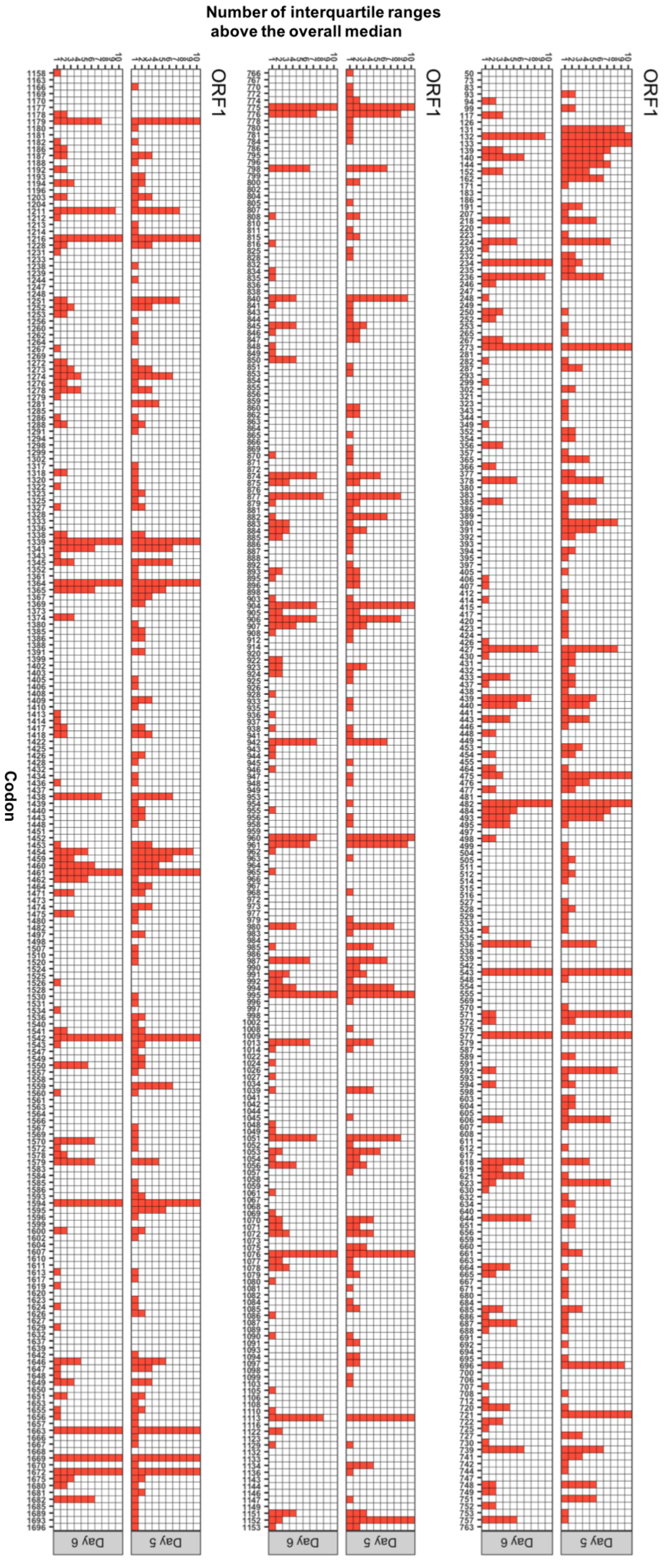

Number of interquartile ranges above the overall median
Codon
ORF1
Day 6
Day 5

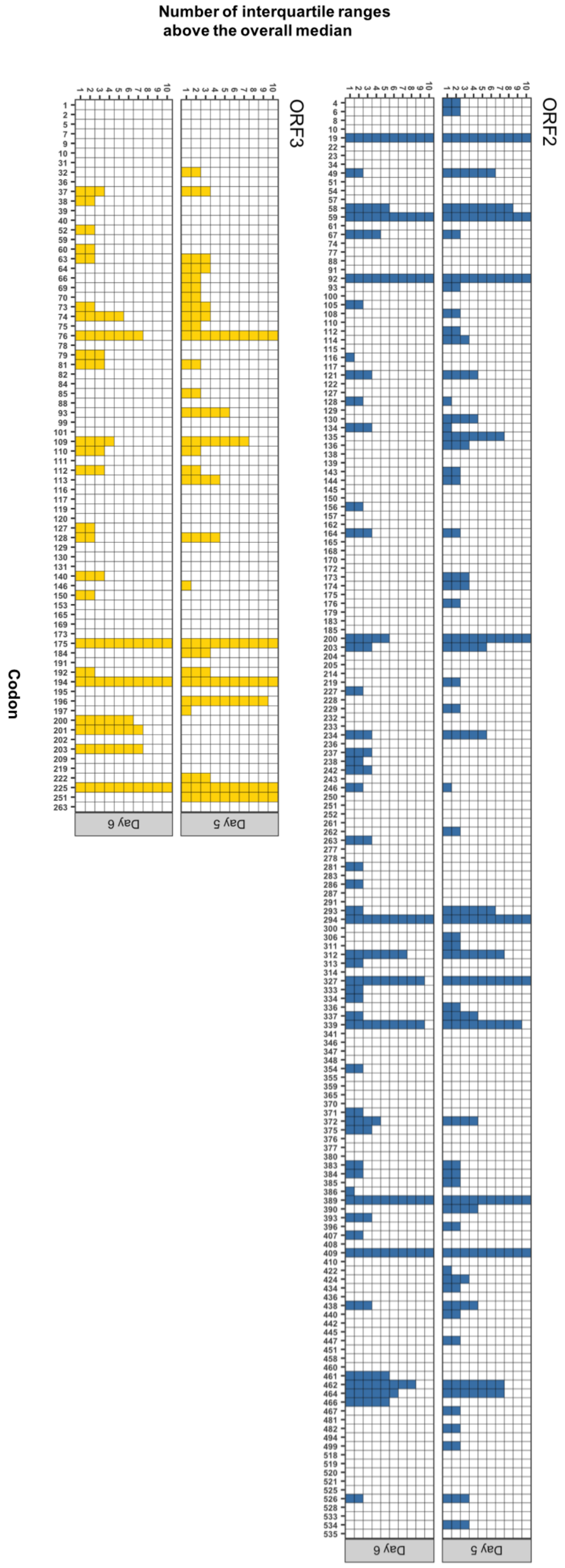
Number of interquartile ranges above the overall median
Codon
ORF3
ORF2
Day 6
Day 5

Supplementary Figure 5

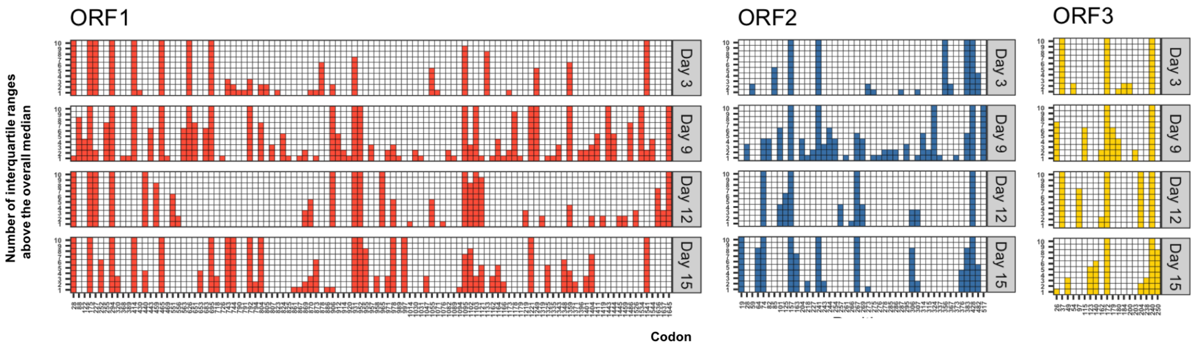

Supplementary Figure 6

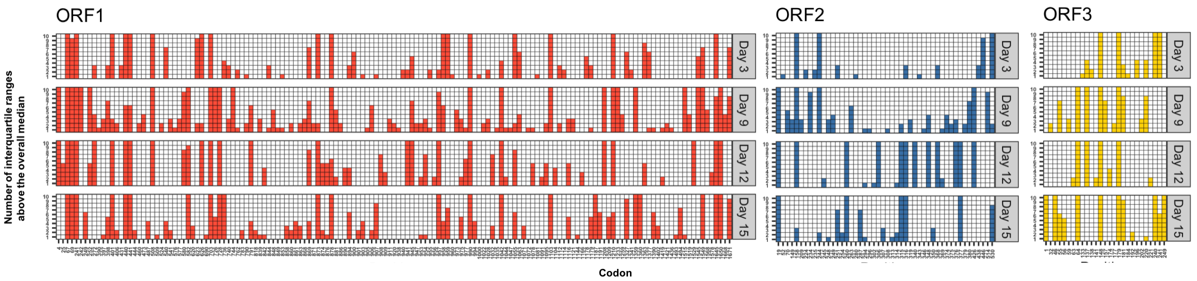

Supplement: Supplementary material 1 [file acmi-3-0203-s001.pdf]
